# Enrichment isolation and metabolic characteristics of *Halobacteriovorax*, cosmopolitan obligate predators in marine conditions

**DOI:** 10.1128/aem.01935-25

**Published:** 2025-11-12

**Authors:** Qian Yu, Feng-Qing Wang, Zi-Yang Zhou, Yu-Qi Ye, Zhenxing Xu, De-Chen Lu, Zong-Jun Du

**Affiliations:** 1 Marine College, Shandong University 12589, Weihai, Shandong, China; 2 Max Planck Institute for Marine Microbiology 28267 , Bremen, Germany; 3 Shandong University Weihai Research Institute of Industrial Technology, Weihai, Shandong, China; Indiana University Bloomington, Bloomington, Indiana, USA

**Keywords:** *Halobacteriovorax*, *Bdellovibrio*-and-like organisms, enrichment isolation, predation dynamics, genomic adaptation, ecological regulation

## Abstract

*Bdellovibrionota,* ubiquitous obligate predators regulating microbial ecosystems, suffer from cultivability limitations with only 12 known isolates despite >1,120 genomes. We developed a prey-addition enrichment system and isolated 33 marine *Halobacteriovorax* strains from sediments that fall into four species, three of which are candidate novel species. Genomic analyses revealed streamlined metabolism with auxotrophy for amino acid/hypoxanthine synthesis, enhanced chemotaxis, and specialized carbohydrate-active enzymes (e.g., GH23 hydrolases), defining adaptations for obligate predation. Predation assays demonstrated broad activity against aquaculture pathogens (*Vibrionaceae*/*Alteromonadales*), optimal at 10² predator:prey ratios. In simulated seawater, isolates achieved a 3-log reduction of *Vibrio alginolyticus* within 10 hours, highlighting potent biocontrol utility. This work bridges cultivability gaps via ecological strategies, advancing evolutionary insights into predation mechanisms while positioning *Bdellovibrionota* as precision biocontrol agents for sustainable aquaculture disease management.

**IMPORTANCE:**

This study resolves the paradox of *Bdellovibrionota*’s ubiquitous genomic presence versus cultivability scarcity by developing a prey-driven enrichment strategy that isolates 33 marine *Halobacteriovorax*. Their streamlined genomes unveil obligate predation adaptations: auxotrophy for amino acid/hypoxanthine synthesis, enhanced chemotaxis systems, and GH23 peptidoglycan hydrolases, illuminating evolutionary trajectories distinct from other predators. Crucially, *Halobacteriovorax* achieves rapid biocontrol, reducing the aquaculture pathogen *Vibrio alginolyticus* by 3 log within 10 hours under marine conditions, outperforming phages in oligotrophic environments. *Bdellovibrionota* shapes the environment by regulating microbial community structures and maintaining ecological balance in ecosystems. Additionally, they hold great promise for being developed as biological agents to replace antibiotics, offering a viable solution for the prevention and control of vibrio diseases in marine aquaculture.

## INTRODUCTION

Bacteria, as the most numerically dominant organisms on Earth, play pivotal roles in biogeochemical cycles and microbial interactions (1). Therein, bacterial predators—defined as microorganisms that actively kill and consume other living cells for energy and biosynthesis—serve as critical regulators for microbial community structures and nutrient recycling through the microbial loop (2, 3). Emerging evidence reveals that bacterial predators also have evolutionary roles in eukaryotic cell origins and multicellularity development (2, 4). These multifaceted impacts underscore the scientific and applied value of predatory bacteria research.

Predatory bacteria exhibit three distinct survival strategies based on metabolic dependencies: (i) obligate predators (e.g., *Bdellovibrio*) require live prey for survival by penetrating prey cell walls to utilize cytoplasmic components (5), and their viability is severely reduced in prey-depleted environments (6); (ii) facultative predators (e.g., *Bradymonabacteria*) exhibit partial metabolic independence and require prey capacity to compensate for deficient biosynthetic pathways (7); (iii) opportunistic predators (e.g., *Myxococcus* and *Lysobacter*) maintain complete metabolic profiles and employ extracellular antimicrobial secretions for predation with independent survival ability in pure culture (4, 8). The functional stratification correlates with specific genomic adaptations, as obligate predators exhibit truncated pentose phosphate pathways and deficient pyrimidine/amino acid synthesis (9, 10), and facultative forms retain partial biosynthetic capacity (7). In contrast to other predators, *Bdellovibrio* demonstrates special predatory activity against high-abundance *Vibrio*, which are primary pathogenic bacteria critically impacting aquaculture industries (11). This ecological interaction highlights the scientific rationale for employing *Vibrio*-targeted enrichment methodologies in the current investigation.

The ecological distinctiveness of bacterial predators becomes evident when compared to phages. Unlike viral predators requiring high concentrations for effective bacterial mortality (12), predatory bacteria demonstrate superior efficiency in low-nutrient environments, earning them the designation of “living antibiotics” (6). This characteristic is particularly valuable in mariculture systems, where *Bdellovibrio* spp. can selectively target pathogens while maintaining microbial equilibrium (13). However, the therapeutic potential of these predators remains constrained by fundamental challenges in their isolation and cultivation.

Despite the documented ubiquity of *Bdellovibrionota* (1,120 genomes representing 719 species across aquatic, terrestrial, and host-associated environments), cultured representatives remain scarce, with only 12 validly published species described to date (GTDB). This disparity between genomic data and cultivability significantly impedes phylogenetic reconstruction and applied research. Our study addresses this critical bottleneck through innovative enrichment techniques that enhance *Bdellovibrionota* abundance, enabling the successful isolation of novel strains. This approach has enabled the first systematic isolation of 33 marine *Halobacteriovorax* strains—a major expansion beyond the previous limit of only 12 cultured isolates across the entire *Bdellovibrionota* phylum. Among these, we describe three candidate novel species, substantially increasing the taxonomic diversity of cultivated predatory bacteria in this group. Subsequent multi-omic investigations, including comparative genomics, metabolic pathway reconstruction, and phylogenomic analysis, provide unprecedented insights into the evolutionary relationships and ecological adaptations of marine *Bdellovibrionota*. Through systematic comparison with other predatory bacterial lineages, we elucidate unique survival strategies of this phylum within microbial predation networks.

## RESULTS

### Enrichment isolation of *Bdellovibrionota*

**Fig 1:**
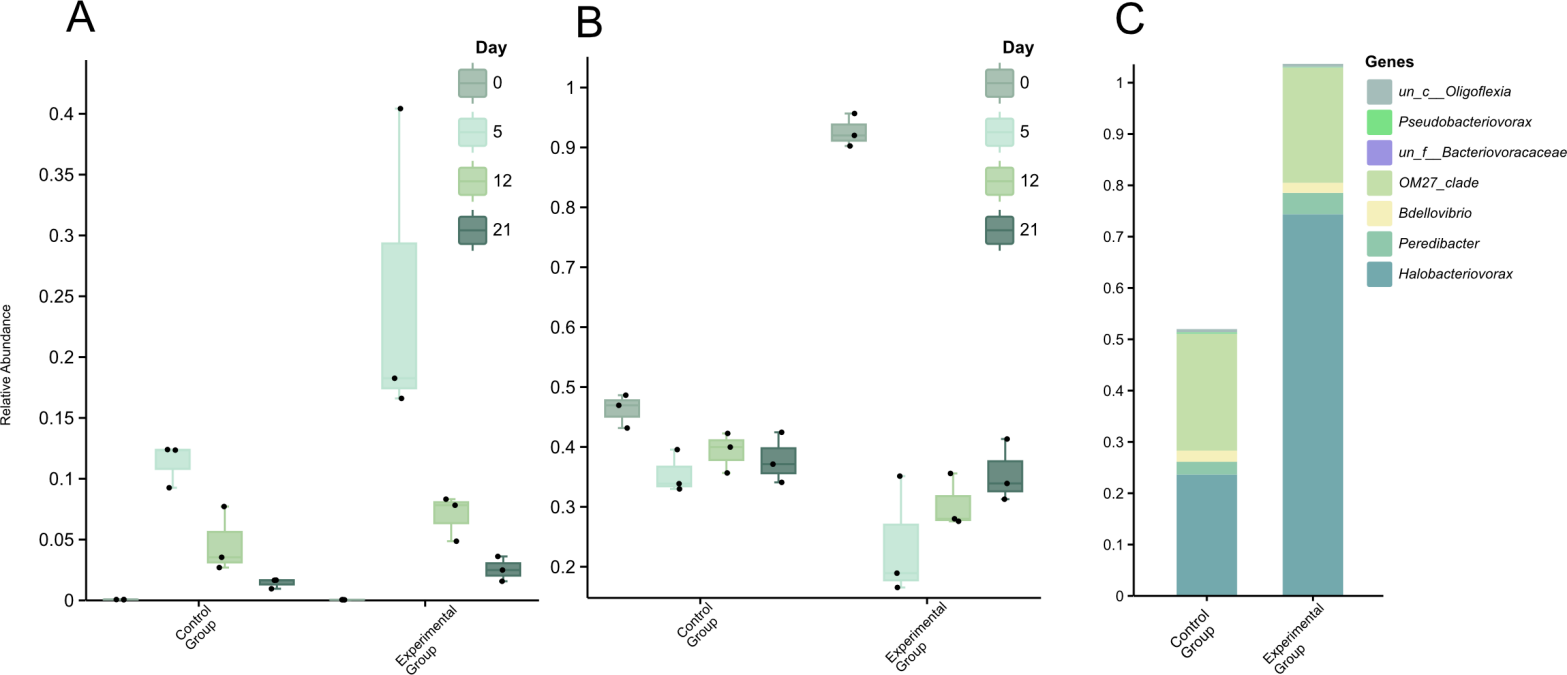
Relative abundance of *Vibrio* and *Bdellovibrio* across times during the enrichment process. (**A**) *Bdellovibrio*. (**B**) *Vibrio*. (**C**) The proportion of different genera of *Bdellovibrio* on the fifth day of enrichment.

Model species were purchased from the Marine Culture Collection of China (MCCC). We constructed a special culture system using the prey bacteria *Vibrio alginolyticus* MCCC 1K03520 to enrich the marine *Bdellovibrionota*. 16S rRNA gene amplicon sequencing analysis revealed that the abundance of *Bdellovibrionota* significantly increased in both the experimental group (supplemented with *V. alginolyticus* MCCC 1K03520) and the control group, with a more pronounced increase observed in the experimental group on the fifth day (Fig. 1). In the experimental group’s enrichment system, the number of *Vibrio* added on day 0 was decreased markedly on the fifth day (Fig. 1B), while the relative abundances of *Bdellovibrionota* were increased significantly (Fig. 1A; Table S1). The results show that *Bdellovibrionota* have the potential to be significant contributors to *Vibrio* mortality, and *Vibrio* provides nutrients for the growth of *Bdellovibrionota*. In such cases, predation by *Bdellovibrionota* may be an important mechanism of nutrient recycling in marine sediments. These results add another dimension to bacterial mortality and the recycling of nutrients. They also provide a method for enrichment culturing of *Bdellovibrionota*.

**Fig 2:**
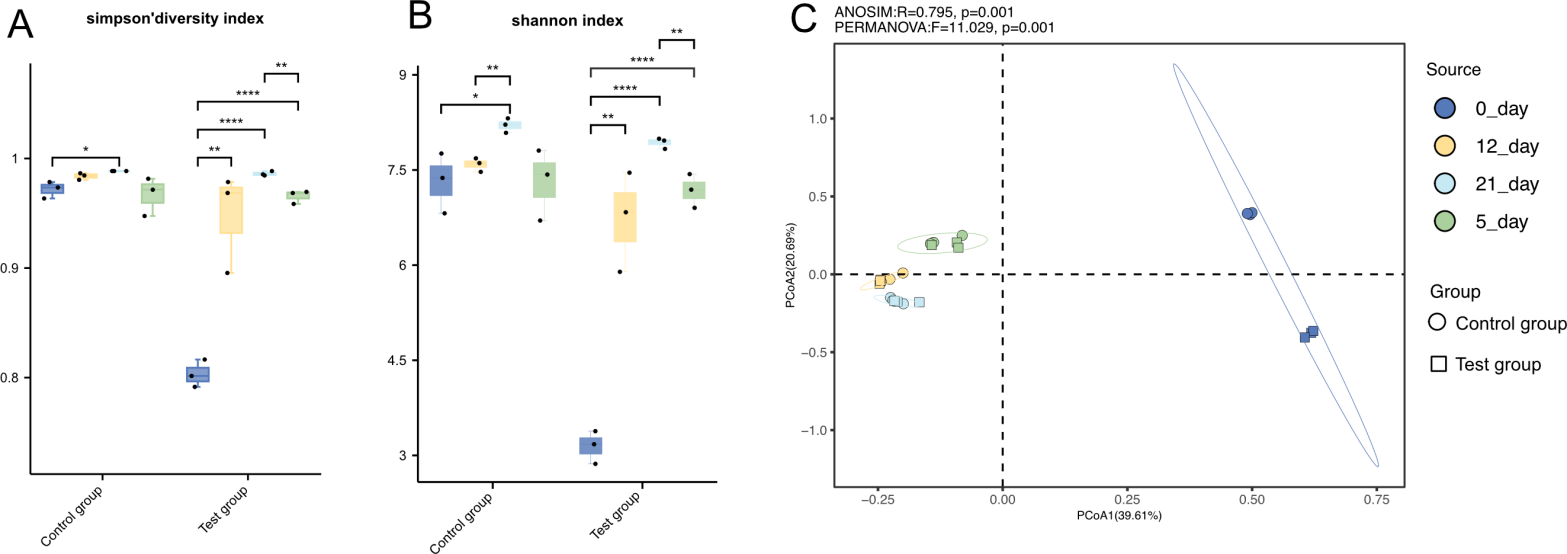
Diversities of control group and experimental group sediment samples as assessed by Shannon, Sini-Simpson indices**,** and principal coordinate analysis (PCoA). Statistical significance was assessed using a pairwise Wilcoxon test with Holm *P*-value adjustment for multiple comparisons (*P <  0.05; **P <  0.01; and ****P <  0.0001). (**A**) Gini-Simpson diversity index. (**B**) Shannon index (α-diversity), paired Wilcoxon test. (**C**) PCoA plots of Bray-Curtis similarities of samples on different days calculated using unweighted UniFrac distances. Each point corresponds to an individual sample (*P* <  0.05; *P* <  0.01; and *P* <  0.001). Details are provided in Table S3.

The Shannon index quantifies the total taxonomic units (species richness) and their relative abundance (evenness) within a sample, serving as a robust metric for α-diversity. High values indicate great community diversity, reflecting both increased species richness and more equitable distribution of individuals among taxa (14). Similarly, the Gini-Simpson index integrates species diversity and evenness, with elevated values signifying heightened community complexity and reduced dominance by a single taxon (15). During the enrichment process, both the experimental and control groups exhibited a biphasic trajectory in the Shannon and Gini-Simpson indices, characterized by an initial increase followed by a decline. The rise in diversity indices corresponds to nutritional supplementation, enabling the transient coexistence of rare taxa in phase 1 (days 0–12). Subsequent decline reflects competitive exclusion and depletion of nutrients, favoring dominance by adapted taxa in phase 2 (days 12–21). Notably, the experimental group, supplemented with *Vibrio*, displayed a significant reduction in α-diversity by day 0, indicative of prey-mediated disruption of the original composition of microorganisms. However, by days 5, 12, and 21 (Fig. 2A and B), the experimental group demonstrated resilience-driven recovery, with diversity indices converging to control levels. PCoA revealed temporal clustering of experimental and control groups at days 5, 12, and 21, with initial divergence (day 5) followed by progressive reconvergence in community structure (Fig. 2C). Considering the abundance changes of *Vibrio* and *Bdellovibrionota*, it is hypothesized that *Bdellovibrionota* plays a significant regulatory role in the homeostasis of the microbial community composition.

During the enrichment process, five clades of *Bdellovibrionota* were detected, and all of them were upregulated. By analyzing the amplicon sequence variants (ASVs) of the *Bdellovibrionota* on the fifth day (Fig. 1C), we found that they mainly belong to *Peredibacter* and *Halobacteriovorax* of the family *Bacteriovoracaceae*, as well as *Bdellovibrio* and gOM27_clade of the family *Bdellovibrionaceae*. In the enrichment system supplemented with prey bacteria, *Halobacteriovorax* was the main group with the highest abundance, reaching 74.3% (Fig. 1C) in *Bdellovibrionota*. Subsequently, we performed target isolation using the samples collected on the fifth day and successfully obtained 33 *Bdellovibrionota* strains, all of which belonged to the genus *Halobacteriovorax* (Tables S4 and S9).

### Identification and predation characterization of new *Bdellovibrionota* members

**Fig 3:**
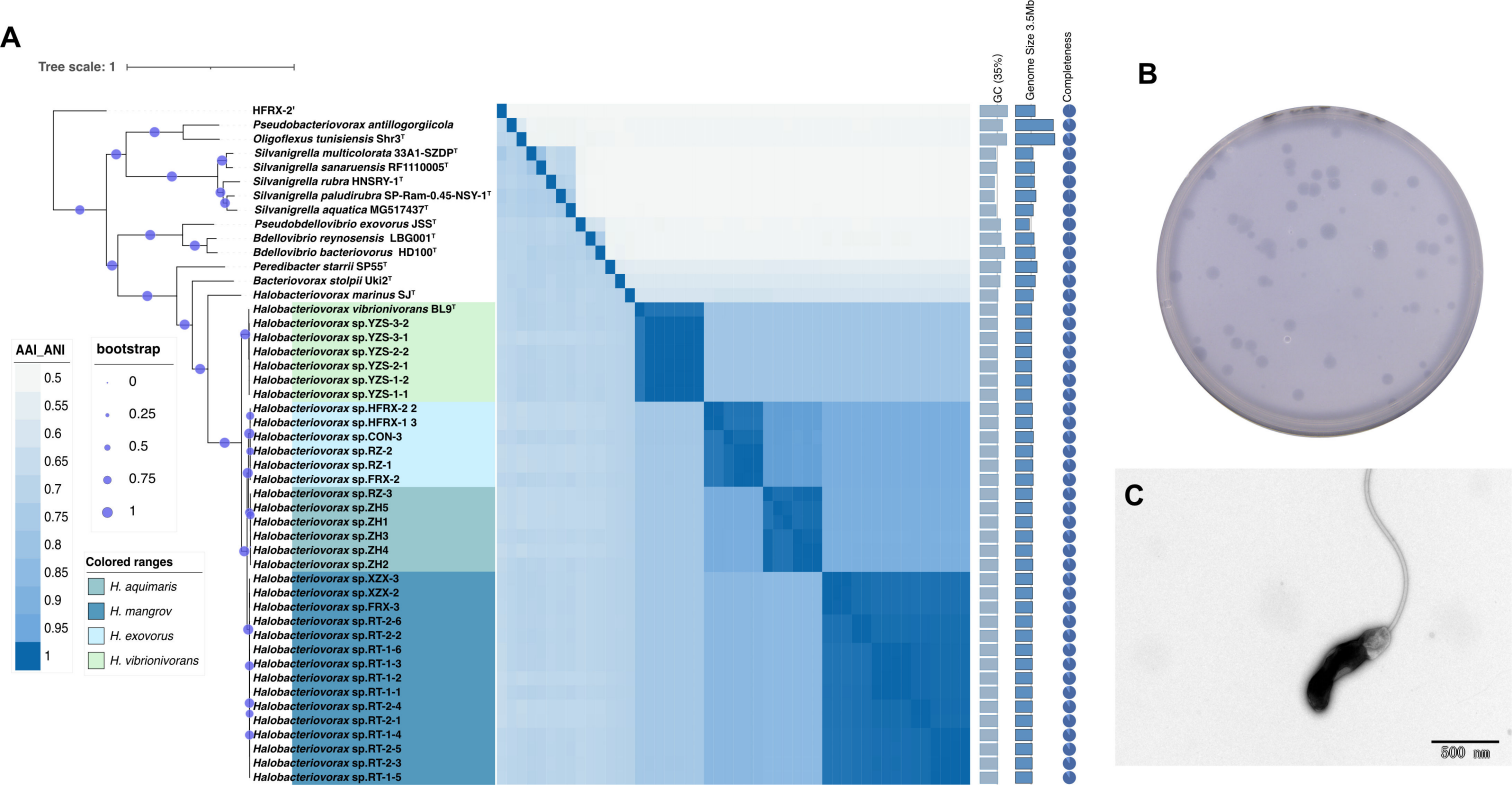
(**A**) Maximum likelihood phylogenetic tree reconstruction based on 120 single-copy marker genes from the genomes of 45 culturable species of the *Bdellovibrionota*. Bar, 0.1 substitutions per nucleotide position. Heat map comparing AAI and ANI values among all bacteria. The lower left part of the figure represents AAI values, and the upper right part represents ANI values. Darker to lighter colors indicate higher to lower values. A darker color indicates a higher value. (**B**) Schematic diagram of the clear zones formed by the predation of *Bdellovibrio* on double-layer plates. (**C**) TEM micrograph of *Halobacteriovorax* sp. ZH4 in pure culture. Bar = 500 nm.

The full 16S rRNA gene sequences of the 33 strains were obtained from their draft genomes and aligned against the NCBI GenBank database using BLASTn. Results showed that these isolates shared 94.5%–100% 16S rRNA gene sequence similarity to the type strain *Halobacteriovorax vibrionivorans* BL9^T^. However, the similarities among these isolates were above 99.8%. Genome sequencing and analysis were performed on the isolated strains with an average raw data of 1 Gb (Table S4). The phylogenomic tree of the culturable strains within the *Bdellovibrionota* revealed that the isolated 33 strains were divided into four independent clusters (Fig. 3A). One cluster belongs to *H. vibrionivorans* BL9^T^, while the other three clusters are potential novel species in the genus *Halobacteriovorax*. The average nucleotide identity (ANI) and average amino acid identity (AAI) were calculated for each pair of strains in the *Bdellovibrionota* (Table S5). As a result, the ANI values within each cluster were above 95%, the AAI and ANI values between three novel clusters and *H. vibrionivorans* BL9^T^ were approximately 78%, and the similarities among the three novel clusters were lower than 95% (Table S5). Given the AAI and ANI values of 95%–96% for species cutoff (16), the 33 isolated strains belong to four species, three of which represent novel species with the proposed names *Halobacteriovorax* sp. ZH4, *Halobacteriovorax* sp. RT1-1, and *Halobacteriovorax* sp. CON-3. This fact also highlights the deficiency of the 16S rRNA gene similarity in species cutoff. Thus, genome-based comparison is necessary for the classification of the *Bdellovibrionota*.

All reported *Halobacteriovorax* strains were isolated from marine environments. Among the 33 isolated strains, *Halobacteriovorax* sp. ZH4 was selected as the representative strain for the following experiments due to its relatively high growth rate. The isolated strains are arc-shaped, possessing a single polar flagellum, and their cell sizes range from 0.3 × 0.5 µm to 1.0 µm, which is much smaller than that of other gram-negative bacteria (Fig. 3B) (17). The isolated strain demonstrated a survival temperature range of 4°C–40°C, with an optimal temperature between 30°C and 35°C. It exhibited the highest predatory activity at 2%–3% NaCl, which aligns with the physiological characteristics of the type strain of this genus. The optimal predatory activity was observed at pH 7.0, showing a high degree of consistency with the optimal pH range of 7–8 reported for *H. vibrionivorans* BL9 (Table S6). The optimal predatory conditions of the isolated strain are highly consistent with the characteristics of the marine habitat.

**Fig 4:**
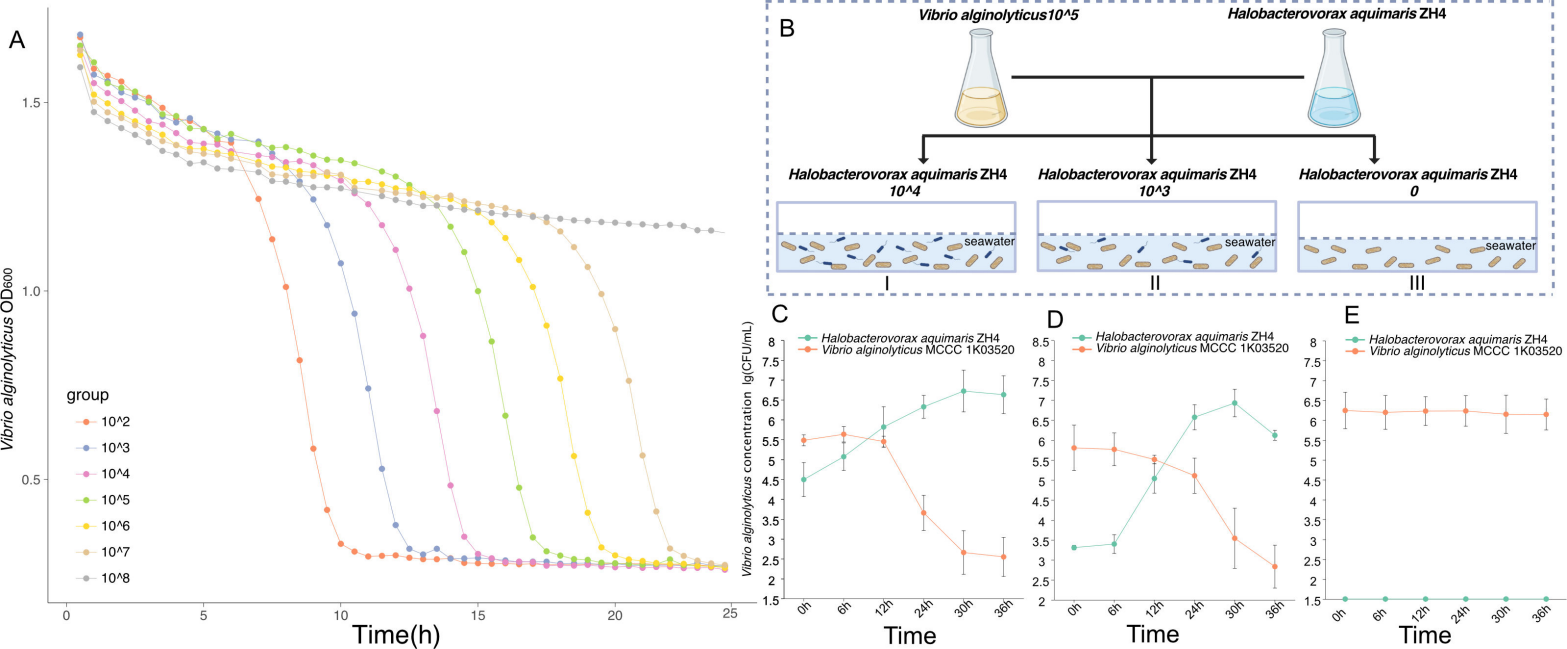
The changes in the predation rate of *Halobacteriovorax* sp. ZH4 at different ratios of predator to prey bacteria. The predator bacterium is *Halobacteriovorax* sp. ZH4, and the prey bacterium is *V. alginolyticus* MCCC 1K03520. (**A**) Changes in the predation rate of *Halobacteriovorax* under aseptic conditions in the laboratory. (**B**) Schematic diagram of the device for simulating open aquaculture environments in the laboratory. (**C–E**) Alterations in the predation rate of *Halobacteriovorax* in simulated open-environment aquaculture systems.

The plaques of the strain are circular, transparent, with smooth and regular edges (Fig. 3C). The predation rate of *Halobacteriovorax* on *V. alginolyticus* depends on the alteration of *Halobacteriovorax* concentration (Fig. 4A). The predation efficiency reaches its peak at the ratio 10² of *V. alginolyticus* to *Halobacteriovorax* and slows down with decreasing *Halobacteriovorax* concentrations. When the ratio of *V. alginolyticus* to *Halobacteriovorax* is 10⁸, the predation of *Halobacteriovorax* disappears (Fig. 4A; Table S7). Moreover, in a laboratory-simulated aquaculture environment containing seawater with a *V. alginolyticus* concentration of 10⁵ CFU/mL, we introduced *Halobacteriovorax* at concentrations of 10³ and 10² CFU/mL into individual non-sealed experimental containers, while maintaining a control group without *Halobacteriovorax* addition (Fig. 4B). Observations revealed that *V. alginolyticus* concentrations declined significantly when supplemented with *Halobacteriovorax*, with the decrease rate being positively correlated to the predator’s initial density and showing faster reduction at 10^4^ CFU/mL than 10^3^ CFU/mL (Fig. 4C and D). In contrast, no concentration change in *V. alginolyticus* was detected in the control group throughout the experimental period (Fig. 4E; Table S8). This finding demonstrates *Halobacteriovorax*’s density-dependent biocontrol efficacy against *V. alginolyticus* under simulated aquaculture conditions. These physiological characteristics of *Halobacteriovorax* enable it to control pathogenic bacteria and maintain the balance of the microbial community in mariculture, laying the foundation for its application in aquaculture.

### Prey spectrum determination of *Halobacteriovorax*

**Fig 5:**
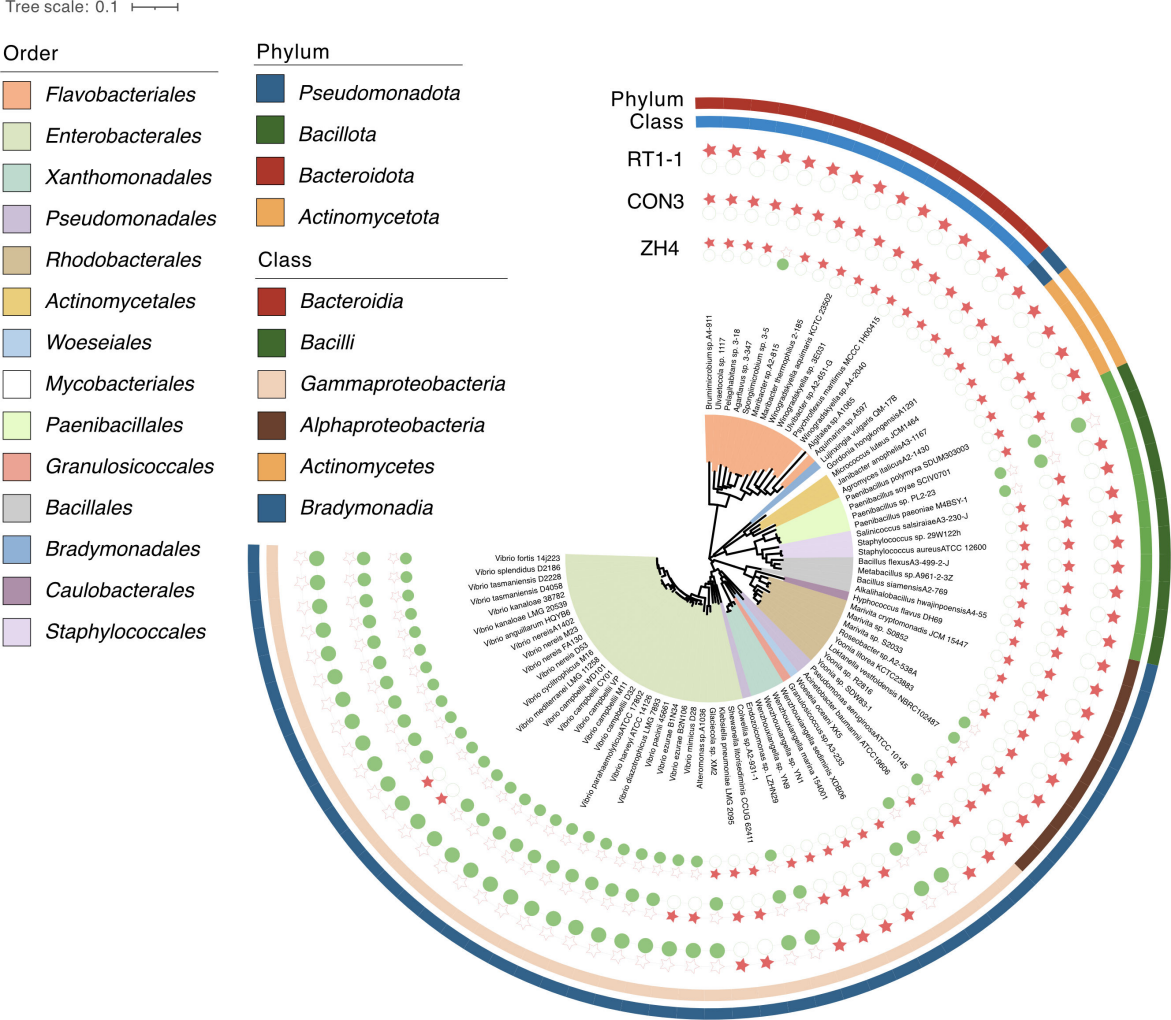
Predation assays for potential prey organisms. A total of 78 bacteria were selected to test predation by one type strain of *Halobacteriovorax*. The phylogenetic tree was constructed for the tested microorganisms. Red dots on the phylogenetic tree indicate that an organism could be preyed upon by strains ZH4, CON3, and RT1-1. Green dots indicate that the organism could not be preyed upon. Detailed information about the microorganisms is listed in Table S9.

*Halobacteriovorax* sp. ZH4 was used to conduct a prey spectrum experiment. As shown in Fig. 5, *Halobacteriovorax* has an obvious predation preference for *Vibrio*, as it can prey on various *Vibrio* bacteria. Strain ZH4 does not infect Gram-stain-positive (G^+^) bacteria, unlike *Bdellovibrio bacteriovorus* HD100^T^, which exhibits predation activity against the G^+^ bacterium *Staphylococcus aureus*, with prolonged survival via an epibiotic mode of interaction (18). The host range of the *Halobacteriovorax* strains isolated in this study is wide (Table S10). For Gram-stain-negative (G^−^) bacteria other than *Vibrio*, *Halobacteriovorax* sp. ZH4 can infect strains in the genus *Alteromonas*, *Woeseia*, *Pseudoalteromonas*, *Shewanella*, *Psychroflexus*, etc. (Fig. 5). Although *Psychroflexus salinarum* ISL-14T and *Psychroflexus maritimus* MCCC 1H00415T both belong to the genus *Psychroflexus*, they exhibit distinct predatory traits. The former can be preyed upon by *Halobacteriovorax*, while the latter cannot. This may be related to the different anti-predation strategies adopted by various bacteria. In addition, to evaluate species specificity, we further determined the prey spectra of the other two candidate novel species, strains CON3 and RT1-1, against a panel of 78 marine isolates (Table S10). Overall, CON3 and RT1-1 also showed broad activity toward G^−^ bacteria and, like ZH4, did not prey on G^+^ bacteria (e.g., *Staphylococcus aureus* ATCC 12600). Prey shared by all three *Halobacteriovorax* included several *Gammaproteobacteria* (e.g., *Pseudomonas aeruginosa* ATCC 10145^T^, *Acinetobacter baumannii* ATCC 19606^T^, *Woeseia oceani* XK5^T^, *Shewanella litorisediminis* CCUG 62411, *Colwellia* sp. A2-931-1, *Wenzhouxiangella sediminis* XDB06, and *Wenzhouxiangella marina* 154001) and *Rhodobacterales* within *Alphaproteobacteria* (e.g., *Yoonia* sp. SDW83-1, *Loktanella vestfoldensis* NBRC 102487^T^, and *Marivita* sp. S0852). Species-specific differences were evident: for example, *Granulosicoccus* sp. A3-233 and *Yoonia litorea* KCTC 23883 were preyed upon by ZH4 and RT1-1 but not by CON3, whereas *Roseobacter* sp. A2-538A was preyed upon by CON3 and RT1-1 but not by ZH4. Within *Marivita*, strain S0852 was susceptible, whereas *Marivita cryptomonadis* JCM 15447^T^ was resistant to all three predators. These data indicate that while the three *Halobacteriovorax* strains are prey generalists among marine G^−^ bacteria, their prey spectra are not identical (see details in Table S10).

### Unique survival strategy of *Bdellovibrionota*

**Fig 6:**
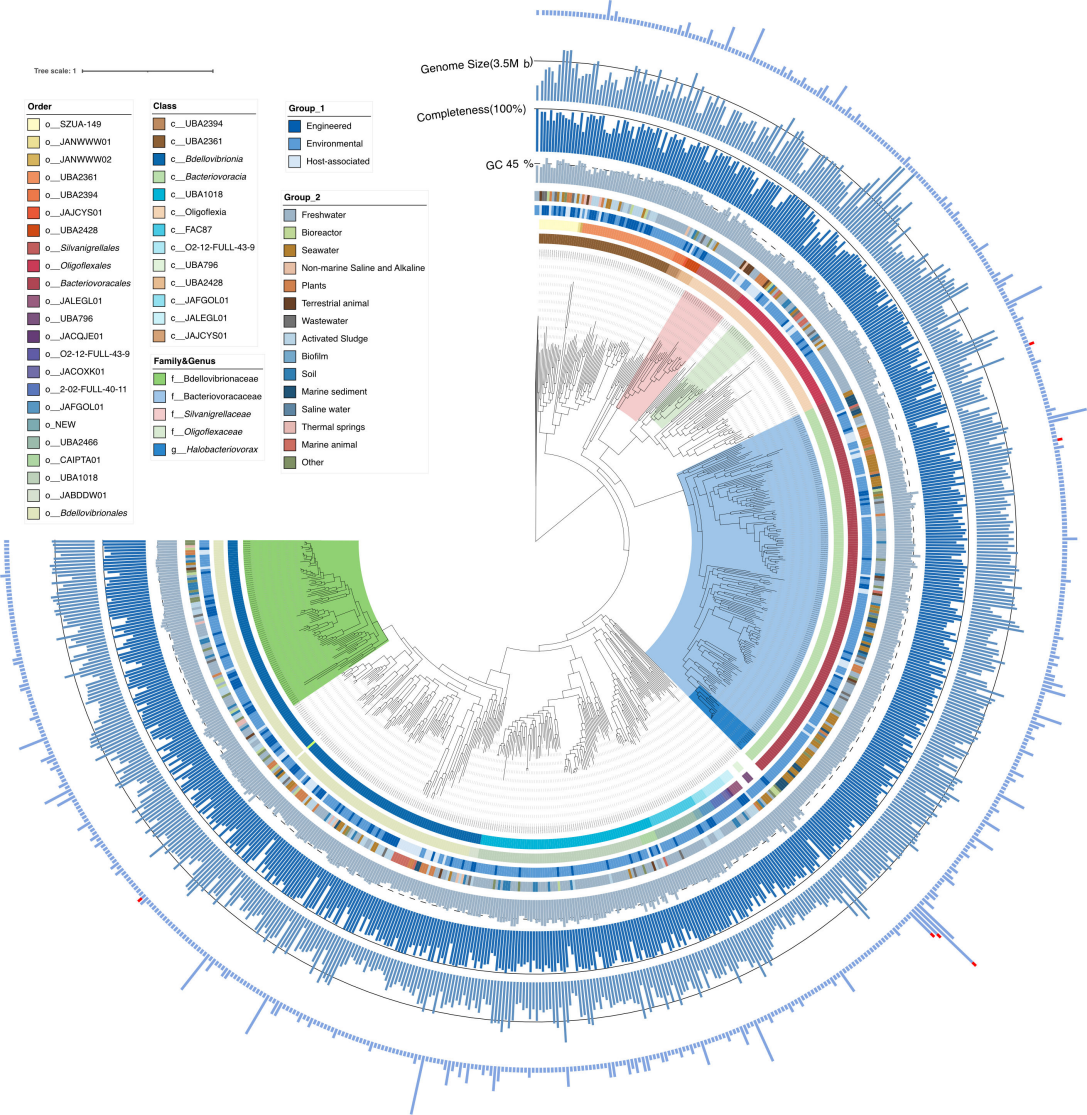
Phylogenomic tree of the *Bdellovibrionota*. Maximum likelihood phylogenomic tree reconstruction based on the 120 single-copy marker genes of 719 genomes of species in the genus *Bdellovibrionota*. Bar, 0.1 substitutions per nucleotide position. Circles from inner to outer are as follows: the taxonomy, ecosystem, ecosystem category, GC content, genome completeness, and genome size. The first and second circles represent the class and order of the *Bdellovibrionota*, respectively. Each color represents a different class or order. The third and fourth circles are the ecosystem classification referring to the JGI GOLD (https://gold.jgi.doe.gov/ecosystem_classification). The legends are arranged in order of quantity. The outer bars represent the number of genomes of the *Bdellovibrionota* before dereplication. After dereplication based on an ANI value of less than 95%, only one genome per species was retained. The red parts indicate the positions of our isolated strains and the MAGs obtained from metagenomes. Bootstraps (≥75) are shown in gray circles.

Based on available genome sequences from public databases (1,120 genomes), there are potentially 719 species (ANI < 95%) in the phylum *Bdellovibrionota*, belonging to 13 classes, 23 orders, and 68 families (Fig. 6; Table S11), including 4 metagenome-assembled genomes (MAGs) of 4 species isolated from our study. However, the number of cultured strains remains extremely limited, with only 12 isolates reported to date. They are mainly distributed across three classes, four orders, four families, and eight genera (Fig. 3). This fact highlights the importance and necessity of isolation and culture of *Bdellovibrionota*. The genome integrity of the 33 newly isolated strains is all above 90%, and the G + C content is between 35% and 37%. The genome size ranges from 3.1 to 3.4 Mb, generally smaller than the average size of bacteria (3.7 Mb) (19), especially smaller than other predation bacteria (5.1 Mb) (Table S12), such as archaeal phylum *Thermoproteota* and nine bacterial phyla (*Actinomycetota*, *Bacillota*, *Bacteroidota*, *Chloroflexota*, *Cyanobacteriota*, *Myxococcota*, and *Omnitrophota*). Notably, *Oligoflexaceae* is a culturable, host-independent *Bdellovibrionota* species that has a significantly larger genome size than other predatory *Bdellovibrionaceae*, *Bacteriovoracaceae,* and *Halobacteriovorax* species (Fig. 6). This fact reflects the evolutionary direction of the predatory *Bdellovibrionota* genomes toward a small size, which is consistent with some parasitic organisms having genomes of smaller size.

**Fig 7:**
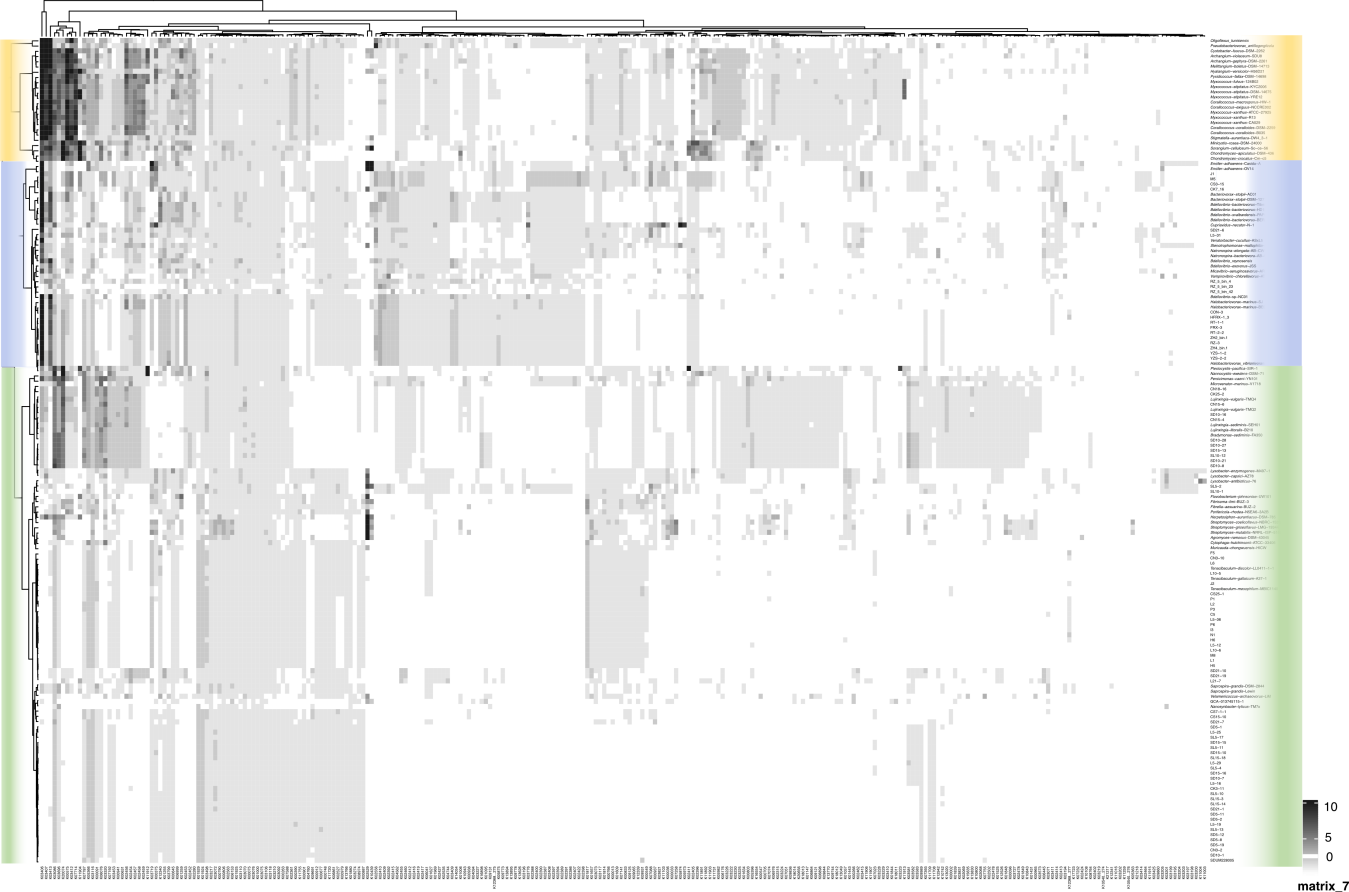
Gene abundance in facultative and obligate bacterial predators. The heatmap was based on a two-way cluster analysis of the genomic abundance of genes encoding KEGG protein groups specific to either facultative predators or obligate predators. Groups with a blue background indicate the so-called obligatory predators. The two-way cluster analysis was clustered using the ward.D2 method based on Euclidean distances. The gene abundance matrix is available in Table S11.

Comparative genomic analysis with other bacterial predators was performed to explore the unique living strategies of *Bdellovibrionota*. Two-way cluster analysis indicated that the genome of *Bdellovibrionota* contains characteristics distinct from opportunists and facultative predators, which were phylogenetically located in a different branch (Fig. 7; Table S13). Meanwhile, we performed a clustering analysis on diverse metabolic pathways, which were denoted by the KO numbers of the enzymes involved in the corresponding reactions as depicted in Fig. 7. This approach was adopted to explore the ecological adaptability of *Bdellovibrionota* as below.

#### Energy metabolism differences

*Bdellovibrionota* shows distinctiveness in the pentose phosphate pathway (M00007). In detail, only a part of *Bdellovibrionota* genomes contain 6-phosphogluconate dehydrogenase (K00033), while this enzyme is more frequent in other predatory bacteria. K03719 is related to the Lrp/AsnC family transcriptional regulator and regulates energy metabolism. K02068 and K02069 are two ABC transport system proteins associated with the ATP binding and permease, respectively, both participating in ATP synthesis. The results of the annotation of these enzymes suggest that *Bdellovibrionota* have different strategies in the energy metabolism pathway to meet the energy demands for predation and growth. Previous studies have demonstrated that *Bdellovibrionota* preferentially uptake extracellular ATP rather than *de novo* synthesis as their energy acquisition strategy (20). This explains the fact that a large number of ABC transporter ATP-binding proteins (K02068) related to ATP transmembrane proteins (K02069) are annotated in the *Bdellovibrionota* strains.

#### Amino acid synthesis differences

*Bdellovibrionota* lacks partial enzymes for synthesizing numerous amino acids, such as *motA* (K02556) involved in lysine synthesis, *metE* (K00549) and *mmuM* (BHMT2, K00547) involved in methionine synthesis. Conversely, other predatory bacteria possess a relatively complete synthesis system (Fig. S11). This indicates that *Bdellovibrionota* needs to acquire a variety of amino acids outside to sustain its growth and metabolism, demonstrating a stronger dependence on the host’s amino acid resources. This is in stark contrast to other predatory bacteria capable of synthesizing multiple amino acids independently.

#### Cell wall synthesis and modification differences

**Fig 8:**
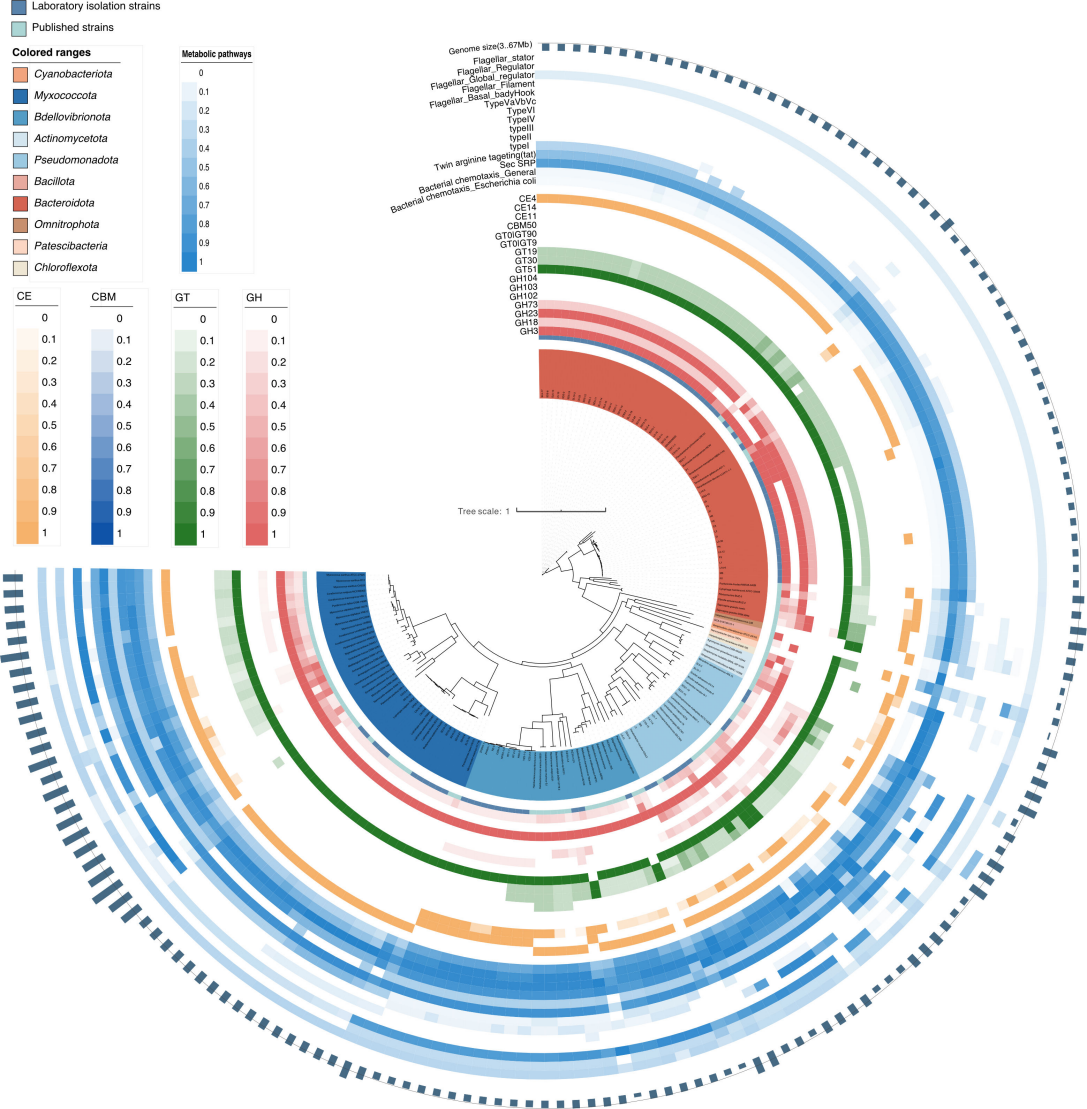
Functional analysis of obligatory predatory characteristics in *Bdellovibrionota*. A maximum likelihood phylogenetic tree was reconstructed based on 120 single-copy marker genes of 163 genomes of all predatory bacterial species identified until 11 November 2024. Bar, 0.1 substitutions per nucleotide position. The tree was modified and visualized using the Interactive Tree of Life (iTOL version 6) (itol.embl.de/). The phylogenetic tree consists of two parts: the first part is a comparative analysis of carbohydrate-active enzymes in *Bdellovibrionota* and other predatory bacteria. The second part is a comparison of the survival strategies between *Bdellovibrionota* and other predatory bacteria. Different phyla of predatory fungi are represented by different label colors.

In terms of cell wall synthesis and modification, *Bdellovibrionota* exhibits high completeness of enzymes related to peptidoglycan synthesis. For instance, the cell wall synthesis and modification-related enzymes *mcmA2* (E5.4.99.2B, K01849) and *mcmA1* (E5.4.99.2A, K01848) have high existence ratios in contrast to other predatory bacteria. This implies the robust ability in cell structures, contributing to cell function and its environmental adaptability. *Bdellovibrionota* retains a highly intact flagellar biosynthesis pathway compared to other predatory bacteria (Fig. 8; Fig. S5). This structural completeness correlates with superior motility, a key trait for prey pursuit in dynamic environments. This indicates more efficient motility and chemotaxis capabilities, which are beneficial for its predation and evasion of predators.

### Functional analysis of obligate predatory characteristics in *Bdellovibrionota*

*Bdellovibrionota* exhibits distinct functional adaptations linked to its obligate predatory lifestyle (Fig. 8; Table S14). (i) Chemotaxis and environmental sensing: *Bdellovibrionota* contains complete chemotaxis pathways (Fig. S3), enhancing precise detection of chemical signals in the environment and facilitating efficient prey localization and adaptive responses during predation. (ii) Secretion systems and biofilm interactions: *Bdellovibrionota* harbors the complete Twin Arginine Targeting (Tat) pathway (Fig. S4A), which supports nutrient acquisition and metabolic waste excretion during predation by facilitating the translocation of folded proteins across membranes (21, 22). Type II secretion system (T2SS) in *Bdellovibrionota* demonstrates significantly higher structural integrity than those of other predatory bacteria. T2SS is critical for toxin secretion and prey interaction (23). Secreted effectors can inhibit metabolic activities of prey bacteria, conferring predatory advantages. Additionally, T2SS-derived proteins contribute to biofilm formation and stabilization, potentially aiding in prey adhesion or predation efficiency (Fig. S4B). (iii) Evolutionary divergence in predatory strategies: comparative analyses reveal metabolic pathway disparities between *Bdellovibrionota* and other predatory bacteria, reflecting divergent evolutionary trajectories (Fig. 7). These differences underscore adaptations in predation strategies, survival mechanisms, and niche specialization, likely driven by selective pressures in distinct ecological contexts. The unique functional profile of *Bdellovibrionota*, including chemotaxis, secretion, motility, and structural biosynthesis, highlights specialized adaptations for obligate predation (Fig. 8). These traits collectively enhance prey targeting, invasion efficiency, and environmental fitness, distinguishing *Bdellovibrionota* from other predatory lineages.

### Comparative analysis of carbohydrate-active enzymes in predatory bacteria

The *Bdellovibrionota* phylum exhibits a significantly lower number of total carbohydrate-active enzymes (CAZyme) than that of non-*Bdellovibrionota* predatory bacteria, with 748 versus 5,176 enzymes, respectively (Fig. 8; Table S12). This stark discrepancy reflects niche-specific adaptations related to obligate predators, since *Bdellovibrionota* primarily exploits host-derived intracellular nutrients rather than environmental polysaccharides, reducing selective pressure for diverse extracellular carbohydrate-processing enzymes (24). Instead, the evolutionary emphasis appears to be placed on predation-related structural and functional specialization. CBM50 facilitates prey recognition by binding surface polysaccharides on target cells, enhancing adhesion and initiating invasion (25). This module enables *Bdellovibrionota* to anchor to prey on surfaces, a critical step preceding enzymatic degradation. GH3 hydrolyzes β-1,4-glycosidic bonds in prey cell wall polysaccharides (e.g., chitin and cellulose), compromising structural integrity (26). GH23 targets peptidoglycan specifically, cleaving the glycosidic backbone of bacterial cell walls to facilitate intracellular access. GT4 synthesizes surface polysaccharides critical for *Bdellovibrionota* biofilm formation and prey interaction. GT51 mediates peptidoglycan biosynthesis, ensuring structural stability during predation-driven mechanical stress. CE4 deacetylates N-acetylglucosamine residues in peptidoglycan, weakening prey cell walls and synergizing with GH23 for efficient lysis (27). Notably, CE4 homologs were absent in these isolated *Bdellovibrionota* strains, suggesting either unannotated paralogs or alternative degradation strategies (Fig. 8).

The streamlined CAZyme repertoire of *Bdellovibrionota* underscores its trophic specialization as an obligatory predator. Reduced investment in extracellular carbohydrate metabolism aligns with energy optimization for host invasion and nutrient acquisition (28). Conversely, retained CAZymes (e.g., GH23 and CBM50) exhibit functional convergence with virulence factors in pathogenic bacteria, highlighting evolutionary parallels in host-cell targeting.

### Ecological distribution of *Halobacteriovorax*

*Halobacteriovorax* strains were detected in various environments, including marine habitats (such as estuaries, salt marshes, marine sediment, seawater, marine animals, and biofilms), terrestrial animals, freshwater, and soil (Fig. S6). Therein, seawater and marine sediment were the preferred habitats, accounting for 50% of the total number of samples. Samples from other saline environments, such as salt marsh and estuary, accounted for 19% of the total. In addition, *Bdellovibrio*-and-like organisms (BALOs) were also isolated from the intestines, surfaces, and other parts of animals, as well as from soil (Fig. S6A; Table S15). The percentages of reads belonging to *Halobacteriovorax* to total reads in different environmental samples were further calculated to estimate the relative abundance for more insights into environmental distribution (Fig. S6). The highest mean relative abundances were in marine environments and salt marshes, especially in marine sediment and marine animals (Table S15). An analysis of the habitat origins of 1,120 genomes obtained from public databases revealed that *Bdellovibrionota* exhibits an extensive distribution range and can be detected in diverse habitats, such as seawater, saline water, freshwater, marine sediments, soil, biofilms, and even in sewage wastewater (Fig. 6). Moreover, they have also been found to colonize the surfaces and intestinal tracts of animals, suggesting they are cosmopolitan obligate predators on Earth.

## DISCUSSION

### Ecological significance of *Halobacteriovorax* in marine systems

Our enrichment experiments demonstrated that *Halobacteriovorax* populations increased significantly in response to *V. alginolyticus* proliferation, correlating with rapid prey mortality (Fig. 1 and 4). This aligns with previous observations that *Bdellovibrionota* acts as keystone predators, regulating bacterial populations and preventing pathogen dominance in low-nutrient environments (2, 6). The density-dependent predation efficiency observed here (Fig. 4A) suggests that *Halobacteriovorax* could act as a natural biocontrol agent in mariculture, reducing *Vibrio* outbreaks without disrupting broader microbial diversity (Fig. 2). The restoration of alpha diversity indices (Shannon and Simpson) post-predation (Fig. 2A and B) further supports their role in maintaining microbial equilibrium, a trait critical for ecosystem resilience (29, 30). Basic beta-diversity analysis suggests that while *Vibrio* supplementation initially disrupts microbial community perturbation, the system exhibits resilience-driven restoration of diversity and structural convergence, likely orchestrated by *Bdellovibrionota* through dynamic predator-prey interactions that recalibrate compositional homeostasis.

The ecological strategy of *Halobacteriovorax* is characterized by euryhaline adaptability and host dependency, suggesting that its growth is not only regulated by salinity gradients but also closely associated with prey abundance and diversity. The distribution patterns of *Halobacteriovorax* suggest that salinity gradients may serve as a key environmental driver for niche differentiation within this genus (Fig. S6A and B). Moreover, strains isolated from host-associated environments, including the hepatopancreas of decapod crustaceans (e.g., *Penaeus vannamei*), digestive glands of bivalves (e.g., *Crassostrea gigas*), and epibiotic biofilms (31), demonstrate highly efficient lytic activity against host-specific vibrios, such as *Vibrio parahaemolyticus* (lysis efficiency > 85%). This observation implies that *Halobacteriovorax* may play dual ecological roles in host-microbe interactions, functioning both as an active predator and a potential symbiont through pathogen suppression. Collectively, the ubiquitous distribution of *Halobacteriovorax* across marine ecosystems, coupled with its dual functional repertoire as a keystone predator, positions it as a critical modulator of microbial equilibrium and a promising candidate for sustainable pathogen management in mariculture systems.

### Metabolic adaptations to obligate predation

The streamlined genomes of *Halobacteriovorax* (3.1–3.4 Mb) reflect evolutionary optimization for parasitism, with significant metabolic deficiencies in amino acid synthesis, pentose phosphate pathways, and cofactor biosynthesis (Fig. 7). *Halobacteriovorax* live in a relatively stable parasitic environment. They do not require complex metabolic and physiological functions like some parasitic bacteria. They lost many genes related to autonomous life, resulting in smaller genomes than other predatory bacteria (32). This is an indication of their adaptation to the parasitic lifestyle, that is, by simplifying the genome to reduce unnecessary energy consumption and utilizing the host’s resources for their own growth. These genomic reductions mirror those seen in other obligate predators (like *Bdellovibrio bacteriovorus*) and underscore their dependence on prey-derived nutrients. Notably, retained pathways, such as flagellar biosynthesis and Tat secretion systems, highlight adaptations for motility, chemotaxis, and toxin delivery (Fig. 7) (2), enabling efficient prey invasion and periplasmic colonization. The absence of CE4 esterases in these isolates suggests alternative lytic strategies, possibly involving synergistic action of GH23 peptidoglycan hydrolases and host-derived enzymes, as observed in *Bdellovibrio* (33).

### Phylogenomic insights and taxonomic implications

The delineation of three novel *Halobacteriovorax* species (*H. aquimaris*, *H. mangrovi*, and *H. exovorus*) based on AAI and ANI thresholds (Fig. 3A) resolves longstanding ambiguities in BALO taxonomy. The incongruence between 16S rRNA gene similarity (94.5%–100%) and genomic divergence (AAI/ANI 78%–95%) underscores the necessity of multi-locus genomic criteria for species classification in *Bdellovibrionota* (34, 35). This aligns with recent calls for genome-based taxonomy in understudied microbial clades (36). The global distribution of *Halobacteriovorax*, particularly in marine sediments and animal-associated niches (Fig. S6), suggests niche specialization driven by salinity tolerance and prey availability, consistent with their predominance in coastal ecosystems (12).

### Biotechnological characteristics and applied potentials

The demonstrated efficacy of *Halobacteriovorax* in reducing *Vibrio* populations under simulated aquaculture conditions (Fig. 4B through E) positions these predators as promising alternatives to antibiotics. However, prey bacteria exhibit a predominant distribution in the *Gammaproteobacteria* (Fig. 5), and the inability to target G^+^ bacteria limits broad-spectrum applications. Engineering lytic enzyme cocktails (e.g., lysostaphin) to destabilize G^+^ bacteria cell walls, as proposed for *Bdellovibrio*, could expand their utility. *Bdellovibrio bacteriovorus* HD100 adopts an “epibiotic” predation strategy against *Staphylococcus aureus*, making direct contact with the cells, and no entry of *Bdellovibrio* into the cells was observed (5, 18). In contrast, it invades the periplasmic space of *Pseudomonas aeruginosa* for predation. These results imply differences in the structures of the two types of bacteria, providing an experimental basis for the speculation that G^+^ bacteria do not have an outer membrane structure similar to that of other G^-^ bacteria (18). Future studies should prioritize field trials to assess predation dynamics in complex microbial communities and investigate horizontal gene transfer risks in open systems (37). Biocontrol risks require consideration but are mitigable: while *Halobacteriovorax* exhibits narrow prey specificity (targeting *Vibrionaceae*/*Alteromonadales* pathogens; Fig. 5) and enables α-diversity recovery post-predation (Fig. 2), non-target effects on beneficial *Gammaproteobacteria* remain possible. Prey resistance may emerge under sustained pressure, though *Halobacteriovorax*’s multi-mechanism physical lysis (GH23/T2SS; Fig. 7) likely delays this compared to single-target antibiotics. As obligate predators, their density-dependent efficacy (10²–10³ PFU/mL for a 3-log reduction; Fig. 4A) poses environmental persistence challenges in open systems. These risks can be mitigated through strain-specific host validation, combinatorial approaches (e.g., phage co-application), and optimized delivery systems.

## MATERIALS AND METHODS

### Sampling information

The original marine sediment samples were collected from Xiaoshi Island (122°6′20″E, 37°32′30″N) in Weihai, China in June 2023; Mangroves (23°55′12″N, 117°25′12″E) in Fujian, China in December 2023; an estuary of Qincun River of Golden Beach Bathing Beach (37°31′55″N 122°3′41″E) in Weihai, China in December 2023; and ballast water from the Admore Cruise Ship. The temperature of the samples was 13°C, the salinity was 21‰, and the pH was 7.6.

### Microbial community analysis

The V3–V4 hypervariable regions of bacterial 16S rRNA genes were targeted for sequencing, as these regions exhibit sufficient sequence variability to resolve taxonomic differences across bacterial taxa (38, 39). PCR amplification was performed using primers 338F (5′-ACTCCTACGGGAGGCAGCAG-3′) and 806R (5′-GGACTACHVGGGTWTCTAAT-3′) to generate amplicons for sequencing. Paired-end reads were assembled into contiguous sequences using FLASH version 1.2.11, which identifies and merges overlapping regions between forward and reverse reads to enhance sequence accuracy (40). Raw data were subjected to quality control with Fastp version 0.19.6, including quality filtering (Phred score ≥ 20), adapter trimming, and removal of reads shorter than 200 bp (41). High-quality sequences were clustered into operational taxonomic units (OTUs) based on a 97% similarity threshold using UPARSE version 11 (42). Taxonomic annotation of ASVs was performed in QIIME2 via the classify-sklearn algorithm, leveraging a pre-trained Naïve Bayes classifier and the SILVA 138.1 reference database (38, 39). Downstream analyses included α-diversity (Shannon index, Chao1), β-diversity (Bray-Curtis dissimilarity), and taxonomic composition profiling (40, 43–45).

The SILVA database was employed to obtain the species classification information corresponding to each ASV representative sequence. To resolve genus-level phylogenetic relationships, representative sequences of the top 100 genera (ranked by relative abundance) were aligned with MAFFT version 7.520 (46) and used to construct a maximum-likelihood phylogenetic tree via IQ-TREE version 2.2.0 (1,000 ultrafast bootstrap replicates) (47). As for *Bdellovibrionota* members, ASVs taxonomically affiliated with *Bdellovibrionota* were manually curated from the data set. Representative sequences of these ASVs were subjected to phylogenetic reconstruction using IQ-TREE version 2.2.0 under the GTR + F + I + G4 model.

### Enrichment, isolation, and purification of BALOs

Approximately 10 g of sediment and 10 mL host bacteria concentrate were added to a sterilized conical flask containing enrichment medium (per liter of seawater: 0.1 g yeast extract and 0.5 g peptone; pH 7.6), and the mixtures were incubated for 0, 5, 12, and 21 days in a shaker (120 r/min) at 30°C. The sediment was collected by centrifugation and stored at −80°C, before sending for high-throughput sequencing and experimental analysis. Non-frozen samples subjected to isolation were homogenized by thorough mixing, followed by serial dilution in a stepwise manner. Aliquots from each dilution gradient were plated onto pre-prepared double-layer plates (preparation protocol detailed in the supplemental material). Inoculated plates were subsequently transferred to a constant-temperature incubator and cultured at 30°C for 2–3 days.

Prey is crucial for the growth and survival of *Bdellovibrionota,* and different *Bdellovibrionota* have distinct preferences for their prey. *Halobacteriovorax* is more likely to prey on marine bacteria such as *Vibrio* (48). Thus, *V. alginolyticus* MCCC 1K03520 was used as the host bacteria for *Bdellovibrionota* isolation. *V. alginolyticus* MCCC 1K03520 was inoculated into 100 mL of 2216E broth and incubated with shaking at 150 r/min at 30°C for 24 hours. The biomass was collected by centrifugation at 8,000 r/min for 10 min. Then, 10 mL of sterile seawater was used to resuspend the biomass, making a host bacteria suspension of ca. 1 × 10^9^ CFU/mL. Alternatively, *V. alginolyticus* MCCC 1K03520 was directly streaked and cultured on 2216E agar plates. The host bacteria suspension of ca. 1 × 10^9^ CFU/mL was prepared using a cotton swab in sterile seawater. To prepare the double-layer plates, 1.2% seawater agar was used as the lower layer medium, while 0.6% seawater agar supplemented with the host bacteria (5:1, vol/vol) was the upper layer medium. The culture and enrichment of prey bacteria *Bdellovibrio* are described in the supplemental material.

### Prey spectrum of *Halobacteriovorax*

The double-layer plate spotting method was used to explore the host range of *Halobacteriovorax*. One milliliter of the host concentrate (ca. 1 × 10^9^ CFU/mL) suspension mixed with the upper agar was poured into the bottom agar plate. Five microliters of *Halobacteriovorax* culture solution was spread on the upper plate and then cultured at 30°C. In addition, the liquid culture method was also used. A volume of 100 µL of the host concentrate (1 × 10^9^ CFU/mL) suspension and 100 µL of *Halobacteriovorax* culture solution were mixed first. Bacterial growth was monitored using a high-throughput real-time microbial growth analysis system (MicroScreen-HT, Jieling Instrument Manufacturing Co., Ltd, Tianjin). One hundred thirty-three bacterial strains used for prey spectrum testing are listed in the experimental materials (Table S9).

### 16S rRNA gene analysis and phylogenomic analysis

The complete 16S rRNA gene sequences were acquired from the individual genome using the ContEst16S algorithm (49). The alignment analysis was performed using the EzBioCloud server (http://www.ezbiocloud.net/) and the NCBI basic local alignment search tool (BLAST) (https://blast.ncbi.nlm.nih.gov/Blast.cgi). One thousand one hundred twenty reference *Bdellovibrionota* genomes were downloaded from NCBI. All selected reference genomes for phylogenetic analysis were sorted out based on the completeness greater than 90% and contamination less than 10% with CheckM version 1.0.5 (50). A total of 676 high-quality MAGs and 43 reference genomes were ultimately incorporated, with their environmental distribution profiles across diverse habitats systematically compiled (Table S10). The concatenated alignment sequences of 120 ubiquitous single-copy proteins were obtained using GTDB-Tk (version 1.3.0) (51), and phylogenomic trees were reconstructed by FastTree (52) with JTT+CAT parameters and IQ-Tree (53) with the LG+F+I+G4 model, both employing 1,000 bootstrap replicates for tree support.

### Genome sequencing and metabolic annotations

The genomic DNA of the 33 isolated strains was obtained from liquid cultures after 72 hours using the Takara MiniBEST Bacterial Genomic DNA Extraction Kit (Takara Bio, Japan). The DNA samples were sequenced by a NovaSeq Sequencer (Illumina, San Diego, CA, USA) with 150 bp PE reads at ≥100× coverage in Beijing Novogene Biotechnology (Beijing, China). Raw data were quality-filtered by BBTools (https://github.com/kbaseapps/BBTools). Assembly of the clean data was performed using SPAdes version 3.9.1 (54). The DNA G + C content was determined from the mean G + C content of the draft genome. Genome statistics of the strains within the genus *Halobacteriovorax* used in this analysis are listed in Table S10. These genomes, along with those of pure culture strains, were subjected to genome dereplication using drep (ANI < 95%). ANI was calculated using pyani (55), while AAI was computed using the AAI calculator (https://github.com/2015qyliang/POCP). Genes were predicted using Prodigal version 2.6.3 (56) and annotated with Prokka (57). Other relevant information for genome evaluation was obtained using CheckM (50). The prediction and annotation of CAZyme genes were conducted following the method described by Lu et al. (58). The specific function of CAZyme genes was searched in Carbohydrate Active Enzymes database (http://www.cazy.org/), and the metabolic pathways were analyzed in detail employing KEGG’s KofamKOALA server (59).

### Biogeographic distribution of *Halobacteriovorax*

The global distribution of *Halobacteriovorax* members was analyzed using the Microbe Atlas Project (MAP, https://microbeatlas.org/, accessed on 30 July 2023). Full-length 16S rRNA gene sequences from each *Halobacteriovorax* genome were submitted to the MAP online server for searching against all 16S rRNA gene amplicon data sets from the NCBI Sequence Read Archive, using a minimum identity threshold of 97%. Samples were removed if the relative abundance of target sequences was less than 10 PPM. The taxonomy of each operational taxonomic unit was identified by vsearch (60) using the SILVA SSU Ref NR 99 138.1 data set. OTUs with more than 97% 16S rRNA gene sequence similarity to members of the genus *Halobacteriovorax* were classified in the genus *Halobacteriovorax*. The habitat distribution of the genus *Halobacteriovorax* was determined by examining the bacterial abundance in global samples, which were referred to the JGI GOLD (https://gold.jgi.doe.gov/ecosystem_classification).

## Data Availability

The genomes of cultured (Table S4) and uncultured *Bdellovibrionota* (Table S11) have been deposited in the NCBI database with the accession numbers listed. The 16S rRNA gene data sets of Xiaoshi Island have been deposited in the Sequence Read Archive under accession number PRJNA1262140 and are also archived at Zenodo (10.5281/zenodo.15471458 and 10.5281/zenodo.17186107).

## SUPPLEMENTAL MATERIAL

Supplemental material aem.01935-25-s0001.docx Supplemental methods; Fig. S1 to S6.

Supplemental tables aem.01935-25-s0002.xlsx Tables S1 to S15.

ASM does not own the copyrights to Supplemental Material that may be linked to, or accessed through, an article. The authors have granted ASM a non-exclusive, world-wide license to publish the Supplemental Material files. Please contact the corresponding author directly for reuse.
