# Supplementary material for: Enrichment isolation and metabolic characteristics of Halobacteriovorax, cosmopolitan obligate predators in marine conditions
Source: Appl Environ Microbiol. 2025 Nov 12;91(12):e01935-25. doi: 10.1128/aem.01935-25 (PMC12724312; doi:10.1128/aem.01935-25)
Supplement: Supplemental material — Supplemental methods; Fig. S1 to S6. [file aem.01935-25-s0001.docx]

Data Article

**Enrichment isolation and metabolic characteristics of *Halobacteriovorax*, cosmopolitan obligate predators in marine conditions**

Qian Yu^1#^, Feng-Qing Wang^3^, Zi-Yang Zhou^1^, Yu-Qi Ye^1^, Zhenxing Xu ^1,2*^, De-Chen Lu^1,2*^ and Zong-Jun Du^1,2*^

^1^ Marine College, Shandong University, Weihai 264209, Shandong, China

^2^Shandong University Weihai Research Institute of Industrial Technology, Weihai 264209, Shandong, China

^3^Max Planck Institute for Marine Microbiology, Bremen 28359, Germany

⁎ Corresponding author

De-Chen Lu, Email: dechenlu@hotmail.com

Zong-Jun Du, Email: duzongjun@sdu.edu.cn

Zhenxing Xu, xuzhenxing@sdu.edu.cn**Supplymentary methods**

***Bdellovibrio* culture on agar plates**

*Bdellovibrio* is diluted to the required concentration gradient, and 100 μL of them were spread onto prepared double-layer plates. The plates were placed in a 30°C incubator for cultivation. When a transparent circle appears, a single transparent circle was selected for liquid culture.

During the preparation of the upper layer medium, the *Bdellovibrio* diluted to the required concentration was directly added. After thorough mixed, the mixture was poured onto the prepared lower layer plate.

***Bdellovibrio* culture in liquid medium**

Add 10 mL of 1×10^9^ CFU/mL host bacteria suspension to 90 mL of sterile seawater. At the same time, inoculate a single transparent circle. Then, incubate with shaking at 150 rpm and 30°C for 24 hours. Perform double-layer plate purification culture more than 3 times until the plaque size is uniform.

**Preparation of *Bdellovibrio* concentrate**

(1) Prepare liquid co-culture of *Bdellovibrio* and the host. When the host bacteria were lysed and the culture medium became clear, we first filtered it with a 0.8 μm filter membrane, and then collected the *Bdellovibrio* cells with a 0.45 μm filter membrane. Finally, centrifuge at 15500×g and 4°C for 30 minutes, resuspend the precipitate with the preservation solution, and store it at -80°C.

(2) Collect the *Bdellovibrio* cells using a 50 mL centrifuge tube. Centrifuge at 2000×g and 4 °C for 10 minutes and transfer the supernatant to a new 50 mL centrifuge tube. Centrifuge the supernatant from the first centrifugation at 5000×g and 4 °C for 15 minutes and transfer the supernatant from the second centrifugation to a new 50 mL centrifuge tube. Centrifuge the supernatant from the second centrifugation at 15500×g and 4 °C for 30 minutes, resuspend the precipitate with the preservation solution, and store it at -80 °C. Using type strain *Halobacterovorax* sp. ZH4 as the experimental control strain, cultivate under optimal conditions.

**Log-Reduction Definition**

A 3-log reduction represents a decrease in the viable microbial population by three orders of magnitude (equivalent to a 99.9% reduction or a 1000-fold reduction), calculated using the formula: log₁₀(N₀/Nₜ) = 3, where N₀ is the initial microbial count and Nₜ is the count after the applied treatment."

**Phenotypic characterization**

To determine the morphological characteristics of the isolates, the strain ZH4 was incubated in marine double-layer plate at 30 ℃ for 3 days. Cell morphology and size were observed using transmission electron microscopy (JEM-1200EX, Jeol). Growth ranges and optima of temperature were determined on double-layer plate at 0, 4, 15, 20, 25, 28, 30, 33, 35, 37, 40 and 45°C. Bacterial growth was tested at different NaCl concentrations (0 to 10%, w/v, in 1% intervals) by adding prey bacteria to artificial seawater (per litre: 3.2 g MgSO_4_, 2.2 g MgCl_2,_ 1.2 g CaCl_2_, 0.7 g KCl, 0.2 g NaHCO_3_). Moreover, the pH range for growth was determined by adding different pH buffers (20 mM) to MB medium (Ye *et al.*, 2024). Bacterial growth was monitored using a high-throughput real-time microbial growth analysis system (MicroScreen-HT, Jieling Instrument Manufacturing Co., Ltd, Tianjin).

**Lysis spectrum of *Bdellovibrio***

To explore the host range of *Halobacteriovorax*, the double-layer plate spotting method is used. After mixing 1 mL of the tested host concentrate (1 mL of 1×10^9^ CFU/mL) suspension with the upper agar, pour it into the bottom agar plate. After it solidifies, draw 5 μL of *Halobacteriovorax* culture solution and spot it on the upper plate. After the upper plate is absorbed, place it in a 30 °C incubator for cultivation. In addition, the liquid culture method is also used. Draw 100 µL of the tested host concentrate (1 mL of 1×10^9^ CFU/mL) suspension and 100 μL of *Halobacteriovorax* culture solution and mix them. Bacterial growth was monitored using a high-throughput real-time microbial growth analysis system (MicroScreen-HT, Jieling Instrument Manufacturing Co., Ltd, Tianjin). A total of 133 strains used for lysis spectrum testing are listed in the experimental materials. Mainly verify it based on the preference for Vibrio, Gram - positive bacteria including Actinomycete, and Gram - negative bacteria other than Vibrio.

**Figure Legends**
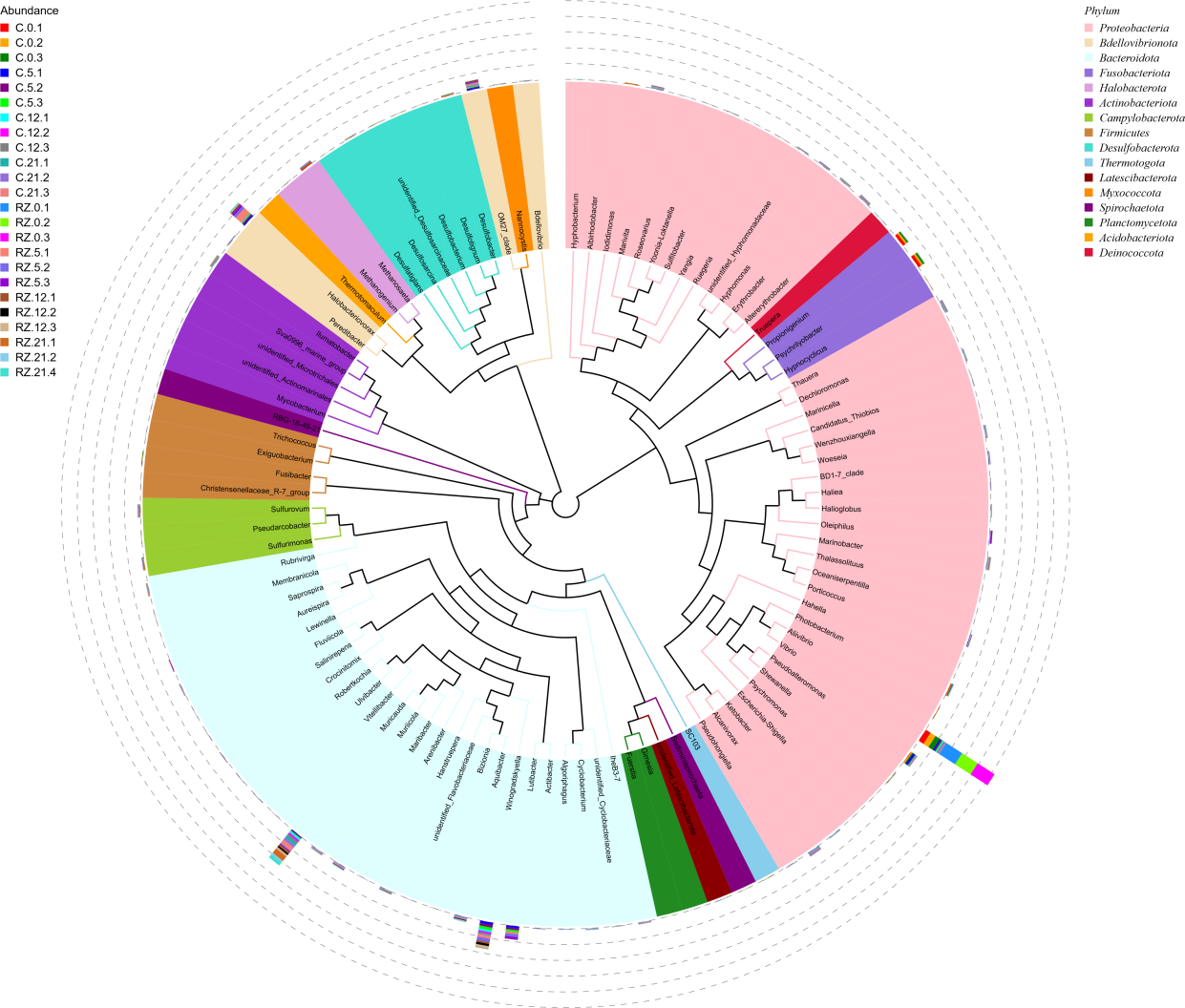


**Fig. S1 The phylogenetic tree constructed from the representative sequences of species at the genus level.** Representative sequences of the top 100 genera were obtained through multiple sequence alignment. Branches and fan sectors are color-coded according to their corresponding phyla, while stacked bar plots positioned outside the fan rings display the relative abundance distribution of each genus across samples. Taxonomic information at the phylum level (right panel) and sample metadata (left panel) are provided as legends for reference.


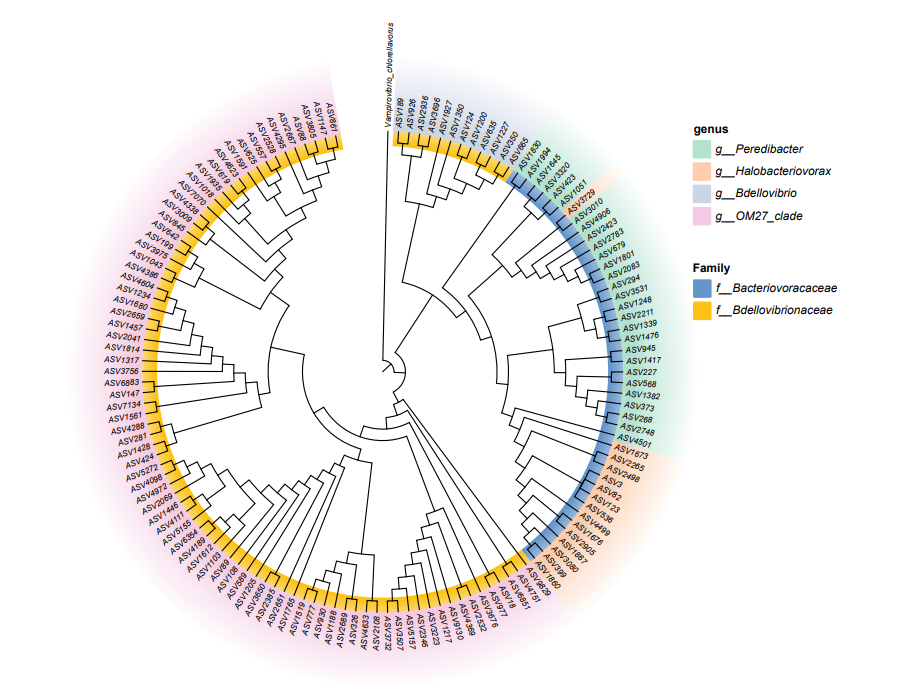


**Fig. S2 Phylogenetic tree of ASVs affiliated with diverse genera within Bdellovibrionota.** ASVs taxonomically affiliated with Bdellovibrio were manually curated from the dataset. Representative sequences of these ASVs were subjected to phylogenetic reconstruction using IQ-TREE v2.2.0 under the GTR+F+I+G4 model


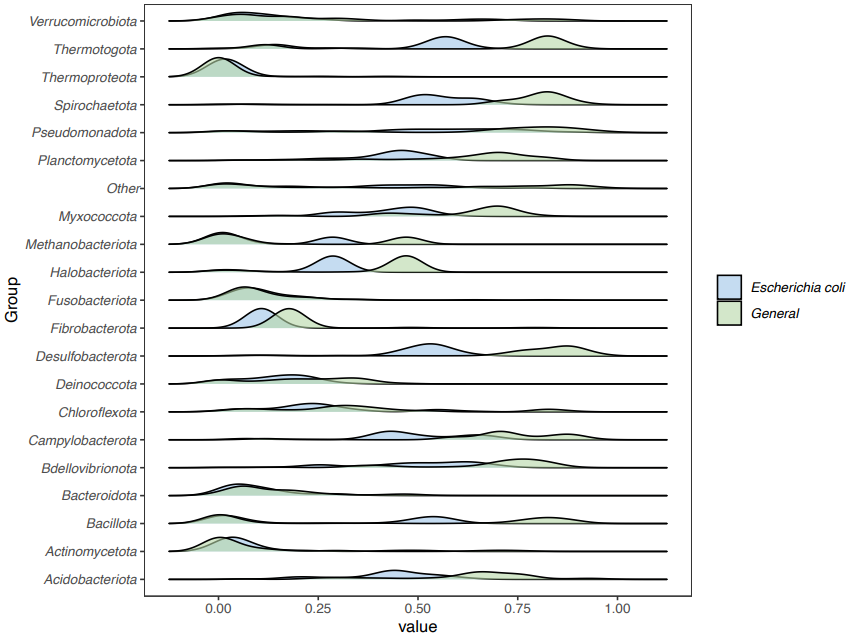


**Fig. S3 Comparative analysis of bacterial chemotaxis pathway completeness across predatory bacterial phyla.** Vertical axis (Group): Represents different phylum - level taxonomic classifications of bacteria, covering various bacterial phyla such as *Verrucomicrobiota*, *Thermotogota*, *Thermoproteota*, etc. Horizontal axis (value): Represents the completeness, with values ranging from 0.00 to 1.00.


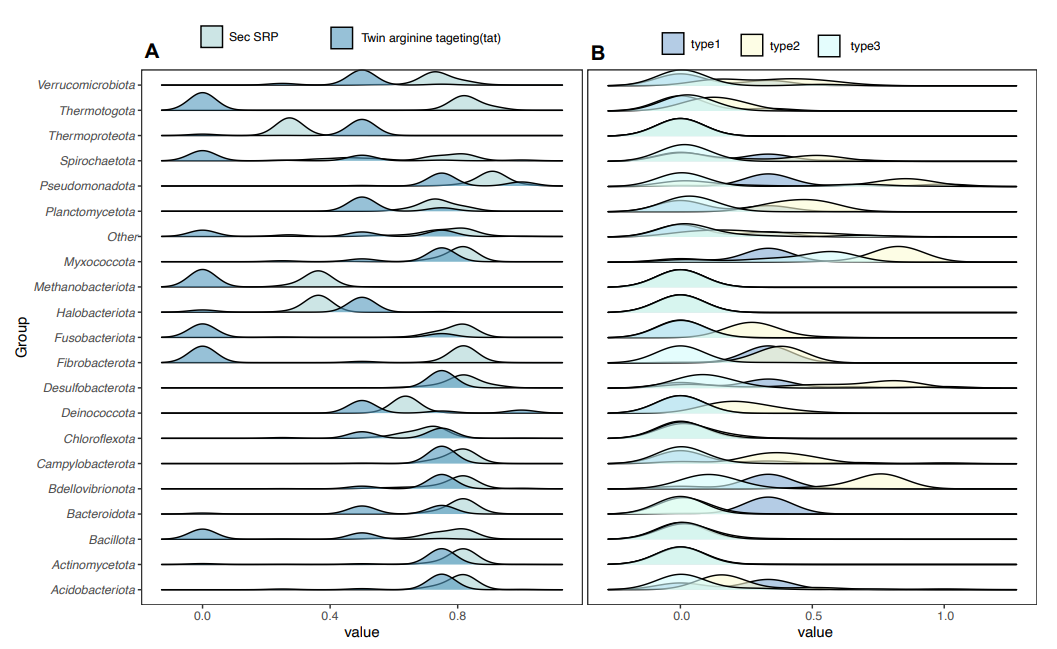


**Fig. S4 (A) Comparative analysis of the secretion system integrity in predatory bacterial phyla. (B) Comparative analysis of the secretion systems integrity in predatory bacterial phyla**. The Sec-SRP is a secretion pathway that depends on the Signal Recognition Particle (SRP). Twin-arginine targeting (Tat) is a secretion system that transports folded proteins. Type 1 is the Type I secretion system, Type 2 is the Type II secretion system, and Type 3 is the Type III secretion system.


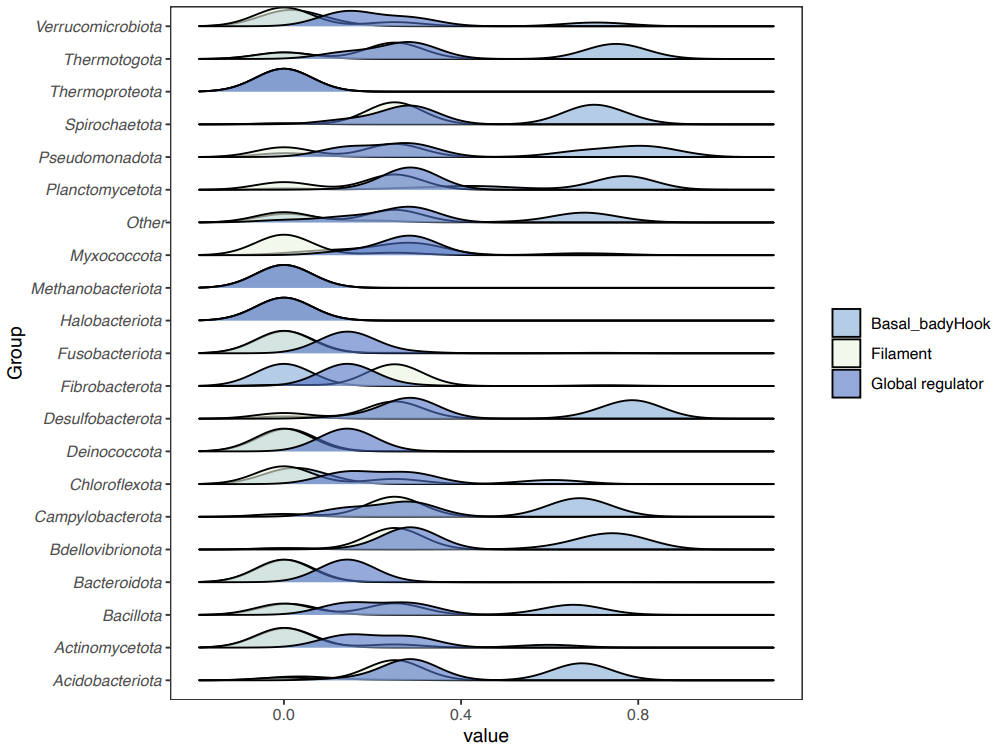


**Fig. S5 Comparative analysis of flagellar completeness across predatory bacterial phyla.**


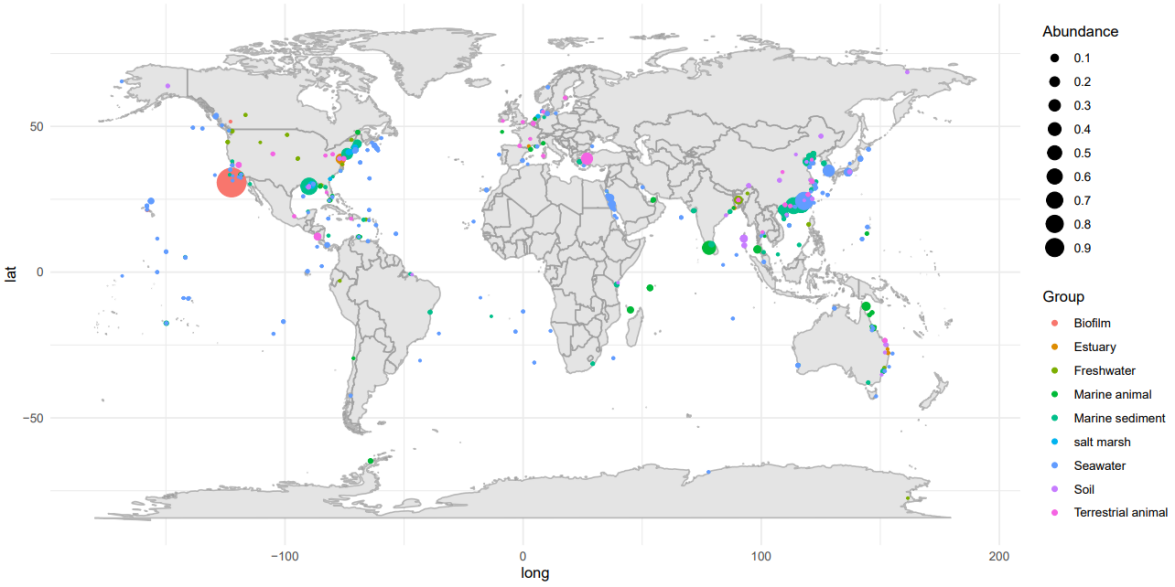

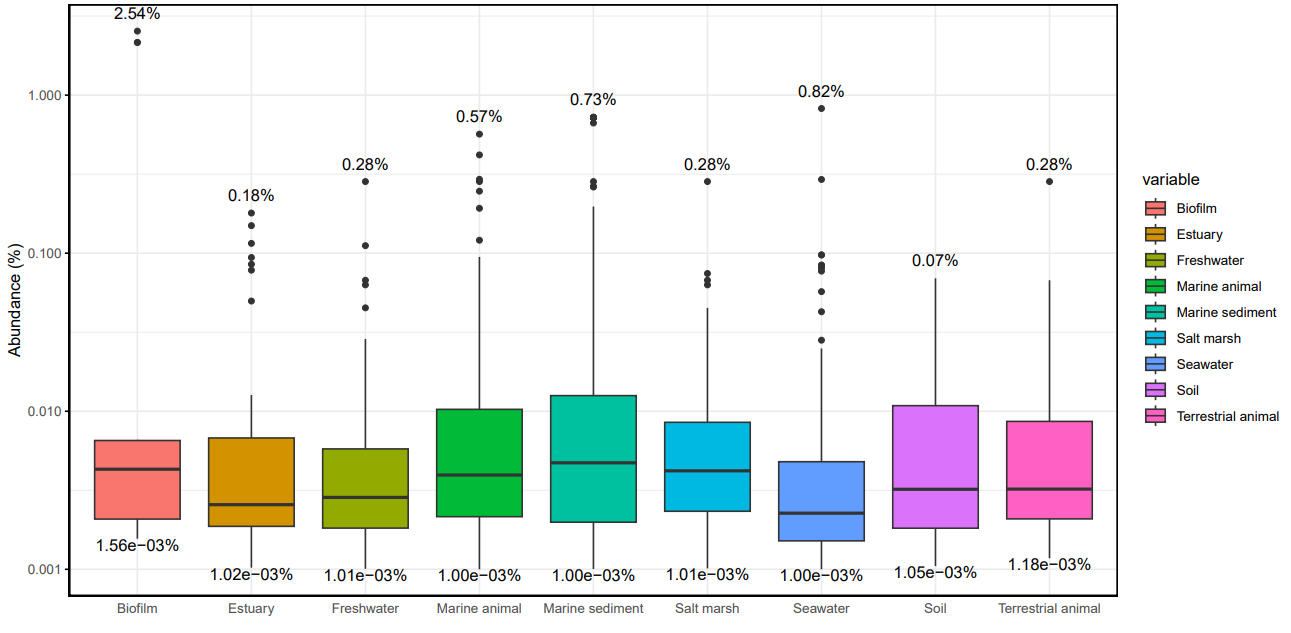


**Fig. S6 The environmental distribution and relative abundance of *Halobacterovorax* based on 16S rRNA gene sequences.** (A) Global distribution of *Halobacterovorax* with relative abundances above 10 ppm. (B) Boxplot illustrating the relative abundances of *Halobacterovorax* across various environments, which were quantified as the percentage of *Halobacterovorax* 16S rRNA gene sequences compared to the total sequence count (Table S14). Values above and below each boxplot indicate the maximum and minimum abundance, respectively. Number of data sets per environment: Biofilm, n=17; Estuary, n=39; Freshwater, n=102; Marine animal, n=227; Marine sediment, n=283; Salt marsh, n=232; seawater, n=330; Soil, n=37; freshwater, n=1,389; Terrestrial animal, n=67.
